# Supplementary material for: Mutual Jellification of Two Bactericidal Cationic Polymers: Synthesis and Physicochemical Characterization of a New Two-Component Hydrogel
Source: Pharmaceutics. 2022 Nov 11;14(11):2444. doi: 10.3390/pharmaceutics14112444 (PMC9692830; doi:10.3390/pharmaceutics14112444)
Supplement: Supplementary file 1 [file pharmaceutics-14-02444-s001.zip › pharmaceutics-1931572-Supplementary.pdf]

pharmaceutics

MDPI

Supplementary Materials

Mutual Jellification of Two Bactericidal Cationic Polymers: Synthesis and Physicochemical Characterization of a New Two-Components Hydrogel

Silvana Alfei [1,*], Alessia Zorzoli [2], Danilo Marimpietri [2], Anna Maria Schito [3], and Eleonora Russo [1,*]

[1] Department of Pharmacy, University of Genoa, Viale Cembrano, 16148 Genoa, Italy

[2] Cell Factory, IRCCS Istituto Giannina Gaslini, via Gerolamo Gaslini 5, 16147 Genoa, Italy

[3] Department of Surgical Sciences and Integrated Diagnostics (DISC), University of Genoa, Viale Benedetto XV, 6, 16132 Genova, Italy

* Correspondence: alfei@difar.unige.it; Tel.: +39 010 355 2296 (S.A.), eleonora.russo@ unige.it (E.R.)

Section S1

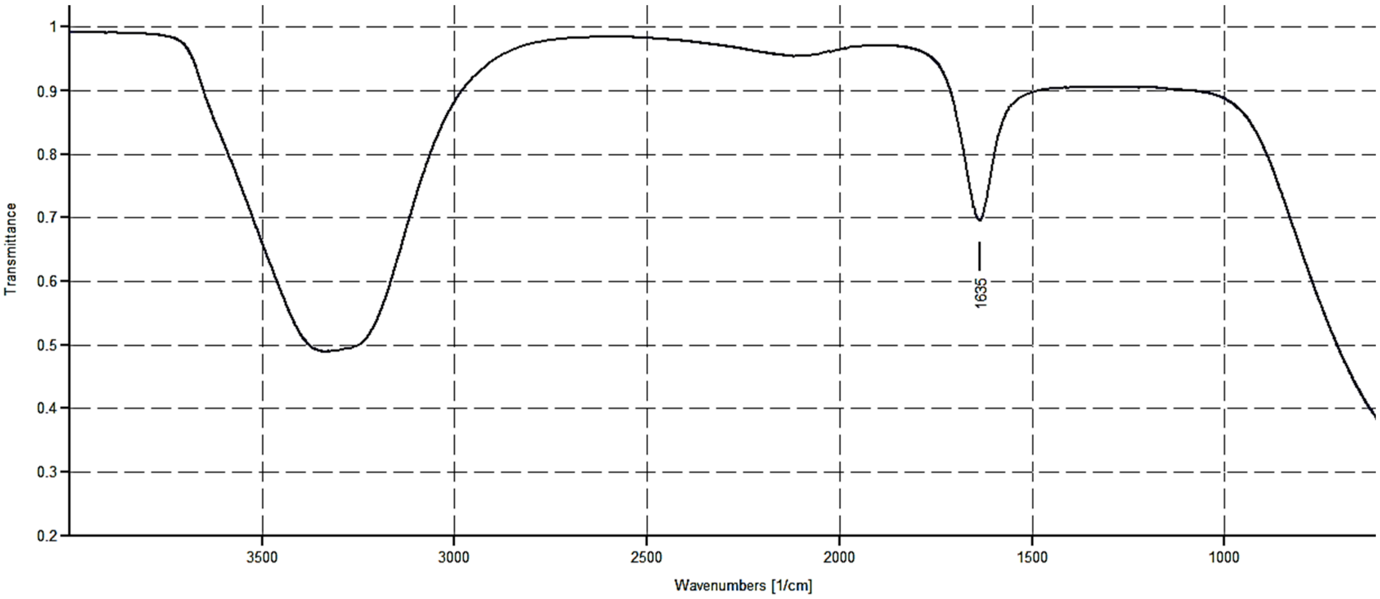

(**a**)

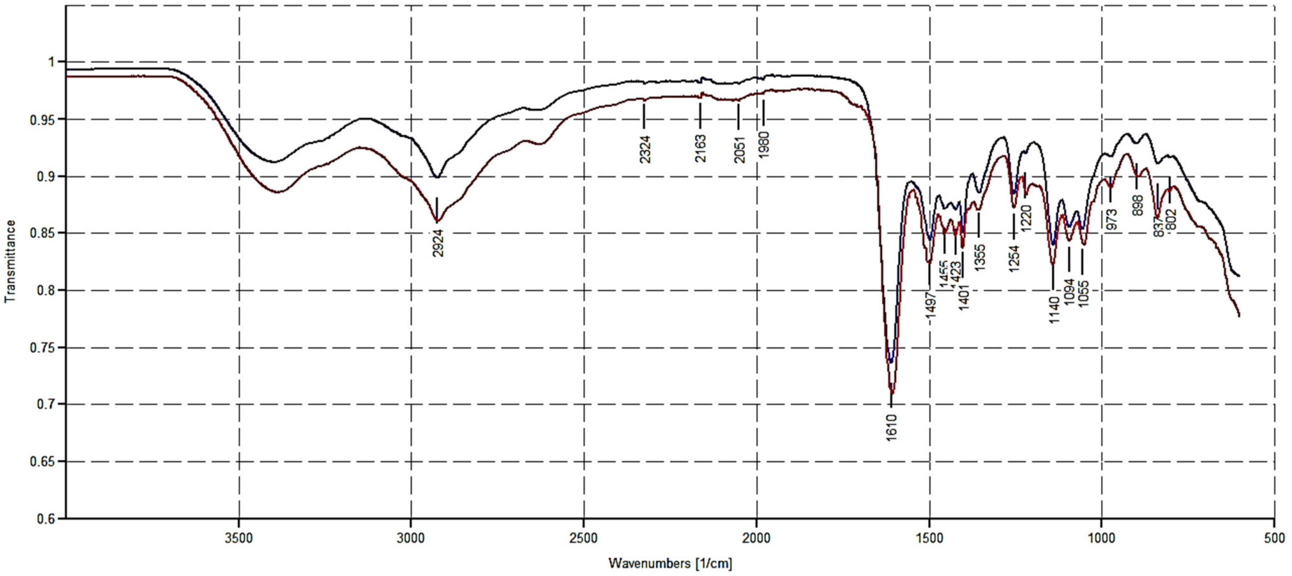

(**b**)

Figure S1. ATR-FTIR spectra of soaked CP1_1.1-Hgel (**a**) and of dryed CP1_1.1-Hgel (dark-blue line) compared with the spectrum of CP1 (red line) (**b**).

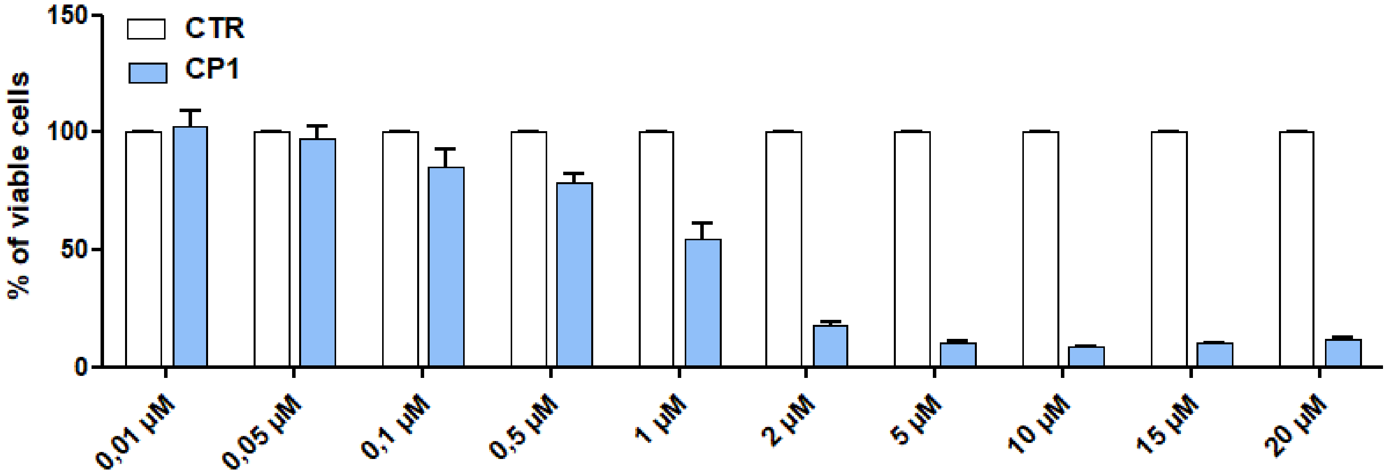

Figure S2. Results from dose-dependent cytotoxicity experiments carried out on MAIL-2 human cells at 4 hours exposure to CP1.

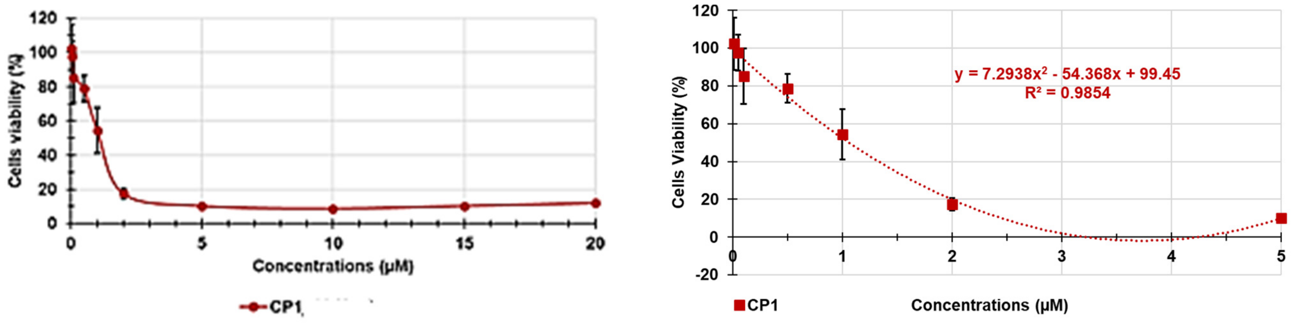

(**a**) (**b**)

Figure S3. Curves of viability (%) of MAIL-2 cells at 4 hours of exposure vs. concentrations (0.01-20 µM) of CP1 (**a**); polynomial tendency line fitting the dispersion graphs obtained reporting in graph the cell viability % vs. the concentration of the sample at 4 hours of exposure (**b**).

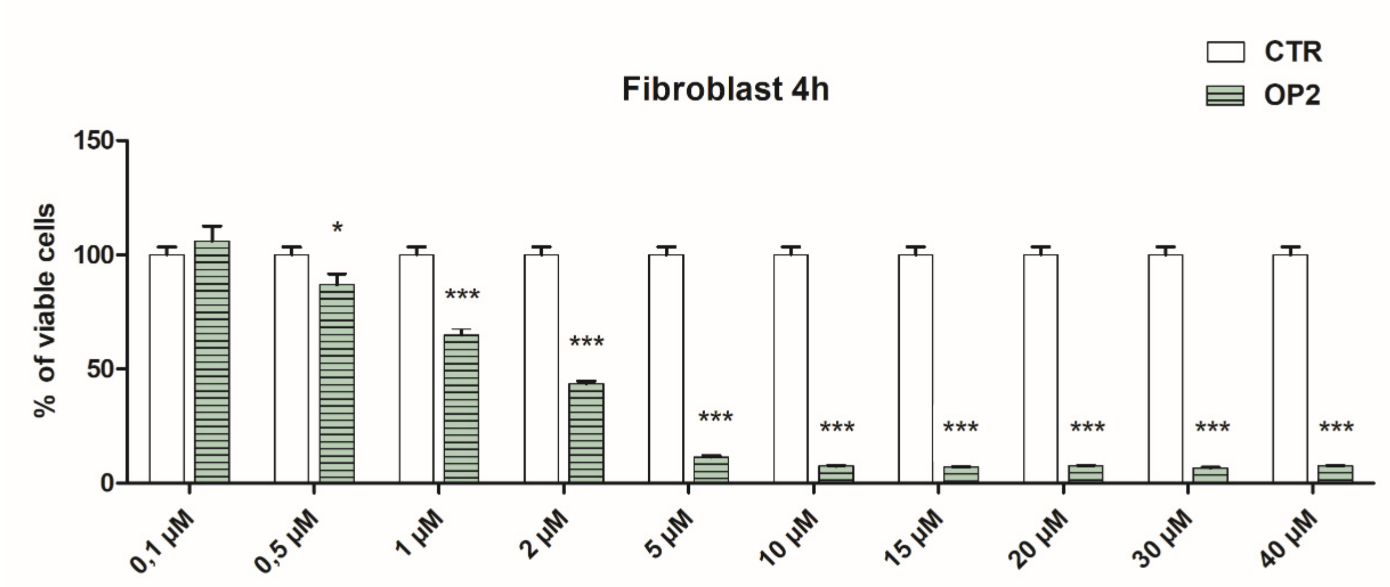

Figure S4. Results from dose-dependent cytotoxicity experiments carried out on MAIL-2 human cells at 4 hours exposure to OP2.

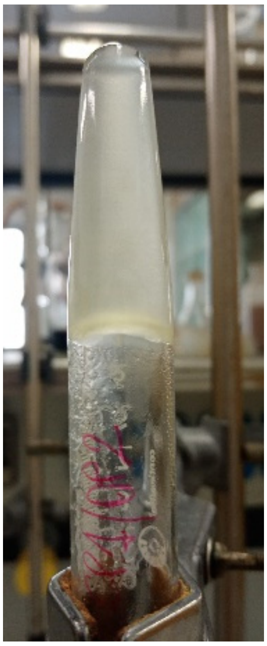

Figure S5. CP1OP2-Hgel obtained as described in Section 2.4 of the associated article, whose volume and weight corresponded to those of gel at its max swelling capability.

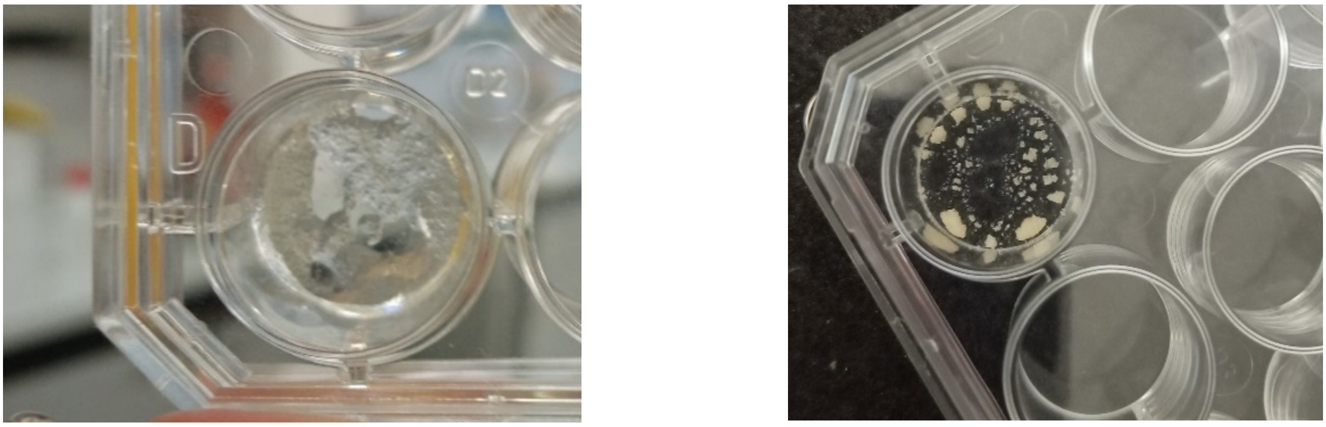

(**a**) (**b**)

Figure S6. Appearance of CP1OP2-Hgel at the time t_0 of the experiments carried out to monitor the water loss in time (**a**); appearance of the fully dried sample (time t_{10}) (**b**).

Table S1. Weights of gel at times T_0-T_{10}, and the computed cumulative weight loss ratio percentages.

**Time (h)**		**Gel (mg)**	**Weight loss (%)**
T_0	0	198.4	0
T_1	0.5	191.5	3.5
T_2	1	164.7	17
T_3	1.5	164.3	17.2
T_4	2.5	142.5	28.2
T_5	3	135.4	31.8
T_6	4	122.0	38.5
T_7	6	84.6	57.4
T_8	7	65.0	67.2
T_9	8	52.9	73.3

T_{10}	24	10.9	94.5

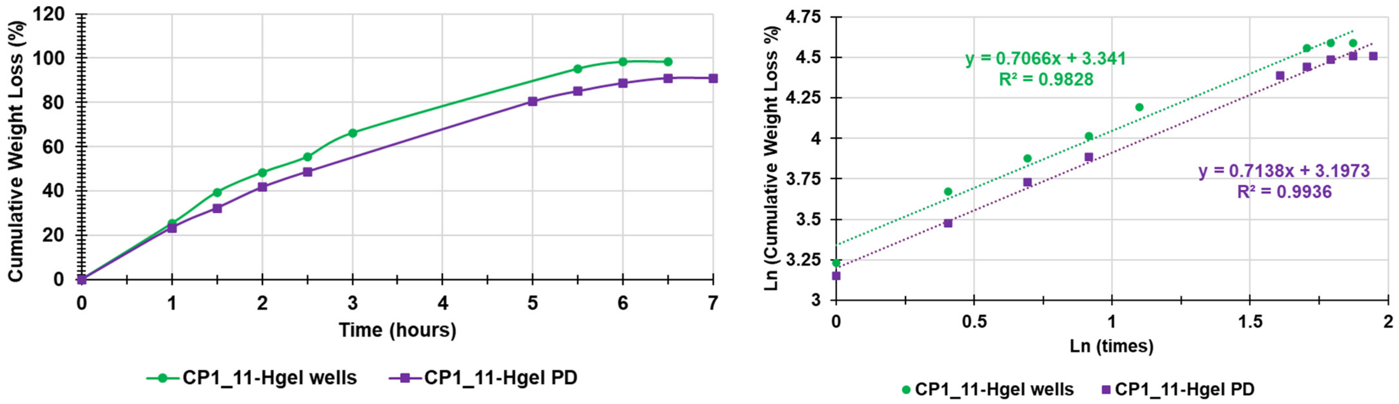

(**a**) (**b**)

Figure S7. Cumulative weight loss percentage curves of gel into Ws (green line) and of that into the PD (purple line) (**a**); Korsmeyer-peppas kinetic models fitting data of cumulative weight loss curves (**b**).

Table S2. Values of coefficients of determination obtained for all the kinetic models used.

Kinetic model	R^2 Hydrogel in Ws	R^2 Hydrogel in PD
Zero order	0.9525	0.9667
First Order	0.9549	0.9906
Hixson-Crowel	0.9525	0.9667
Higuchi	0.9780	0.9777
**Korsmeyer-peppas**	**0.9828**	**0.9936**
